# Retinopathy-associated inosine monophosphate dehydrogenase 1 mutations cause metabolic and filament defects in cones

**DOI:** 10.1242/dmm.052389

**Published:** 2025-09-17

**Authors:** Kaitlyn M. Rutter, Michelle M. Giarmarco, Vivian Truong, Yekai Wang, Mark Eminhizer, Yinxiao Xiang, Whitney M. Cleghorn, Gardenia Sanchez, Anika L. Burrell, Justin M. Kollman, Jianhai Du, Susan E. Brockerhoff

**Affiliations:** ^1^ Department of Biochemistry, University of Washington, Seattle, WA 98195, USA; ^2^ Department of Ophthalmology, University of Washington, Seattle, WA 98195, USA; ^3^ Department of Biochemistry and Molecular Medicine, West Virginia University, Morgantown, WV 26506, USA

**Keywords:** IMPDH1, Retinitis pigmentosa, Purine metabolism, Retina, Zebrafish

## Abstract

Dominant variants in inosine monophosphate dehydrogenase 1 (IMPDH1), a key enzyme in the *de novo* synthesis of purine bases, cause progressive photoreceptor death, leading to blindness. To investigate the cause of degeneration, we generated the first mutant IMPDH1 animal models and expressed mutant forms of *impdh1a* in zebrafish cone photoreceptors. Unlike cones expressing exogenous normal *impdh1a*, cones containing *impdh1a* with the K238E mutation degenerated. Cones expressing *impdh1a* with the D226N mutation did not show significant cone loss by 2 years. Steady-state and flux metabolomics in zebrafish retinas revealed no differences in glucose shunting to the pentose phosphate pathway, no change in AMP or GMP due to D226N expression, but reduced AMP/IMP and GMP/IMP in K238E-expressing cones. cGMP levels were normal in both mutant retinas. Further, *pde6c^w59^*; *impdh1a^sa23234^* double mutant cones were not rescued from degeneration. Both K238E and D226N mutant-containing proteins formed abnormally large mislocalized filaments, which could disrupt normal dynamic protein–protein interactions. Our work disproves the model of a hyperactive enzyme leading to elevated cGMP causing cell death and reveals new defects associated with IMPDH1 mutant expression.

## Teaser

**Summary:** In a zebrafish model of cone degeneration, Impdh1a class 1 mutations do not cause elevated cGMP but do cause alterations in steady-state metabolism and filament size and localization.

## INTRODUCTION

Autosomal dominant retinitis pigmentosa and Leber congenital amaurosis are photoreceptor degenerative diseases that lead to blindness. Mutations in several proteins, including inosine monophosphate dehydrogenase 1 (IMPDH1), a key enzyme in the *de novo* purine biosynthesis pathway (Burrell and Kollman, 2022; Sakti et al., 2023), cause these diseases. IMPDH1 is abundant in both rod and cone photoreceptors (Cleghorn et al., 2022; Karlsson et al., 2021; Plana-Bonamaisó et al., 2020), and, unlike the typical autosomal dominant retinitis pigmentosa phenotype, whereby rods die first and cones secondarily die, patients with IMPDH1 mutations often have an early cone defect (Bennett et al., 2020; Sakti et al., 2023; Wada et al., 2005; Wendel et al., 2024).

IMPDH1 sits at a branch point between adenine and guanine synthesis, where it converts inosine monophosphate (IMP) into xanthine monophosphate (XMP), reducing NAD^+^ to NADH. IMPDH1 forms octamers that can stack to form filament structures in cells. It can be allosterically regulated by downstream products, ATP and GTP (Buey et al., 2015; Fernández-Justel et al., 2019a,b). These filaments can interact with other metabolic proteins involved in purine or pyrimidine metabolism for further regulation (Pedley and Benkovic, 2017). The retina has a specific IMPDH1 isoform that is less sensitive than the canonical isoform to GTP inhibition (Burrell et al., 2022). Zebrafish photoreceptors predominantly express a specific *impdh1a* transcript (*tvX1*) (Cleghorn et al., 2022). The encoded variant has similar structural and functional characteristics to human retinal IMPDH1 (Cleghorn et al., 2022).

Purine metabolism is essential for nucleotide production, which is necessary for DNA and RNA synthesis, energy currency including ATP and GTP, and production of signaling molecules, such as cGMP. Photoreceptor energy demand is uniquely high – a mouse rod photoreceptor is reported to consume 10^8^ ATP/s, which is used to maintain the membrane potential (Okawa et al., 2008). Photoreceptor outer segment membranes are packed with proteins required for phototransduction. Rhodopsin, a protein responsible for initial light detection, has ∼60,000,000 molecules per mouse outer segment (Skiba et al., 2023). Ten percent of mouse rod outer segments are renewed daily (Umapathy et al., 2023; Young, 1967). cGMP is essential for phototransduction in photoreceptors. Phosphodiesterase 6 (PDE6) is an enzyme in photoreceptors that hydrolyzes cGMP to GMP in the light, which allows cyclic nucleotide gated (CNG) channels to close (Arshavsky et al., 2002). PDE6 mutations can increase cGMP levels, leading to photoreceptor death (Farber and Lolley, 1974; Power et al., 2020). Photoreceptor degeneration stemming from a nonsense mutation in the *Pde6b* gene can be delayed by treating mice with an IMPDH inhibitor (Yang et al., 2020).

The mechanism behind IMPDH1-related degeneration is unknown. Knocking out Impdh1a has no impact on zebrafish photoreceptor structure, and mice with IMPDH1 knockout have a mild retinopathy (Aherne et al., 2004; Cleghorn et al., 2022). This suggests that mutations in IMPDH1 that cause degeneration cause a gain of function. Further, several mutations are clustered near ATP and GTP binding sites (Burrell and Kollman, 2022; Sakti et al., 2023), and a subset of these (termed class 1) mutations disrupt the inhibitory allosteric regulation of the protein by GTP (Burrell et al., 2022; Burrell and Kollman, 2022). One hypothesis is that, *in vivo*, hyperactive IMPDH1 could cause elevated levels of downstream guanine nucleotides, including cGMP, which would promote cell death. However, evaluating IMPDH1-associated photoreceptor cell death has not been possible owing to the lack of IMPDH1 mutant animal models.

Here, we present the first IMPDH1 mutant disease models consisting of zebrafish expressing the class 1 D226N or K238E mutation specifically in cone photoreceptors. We focused our studies, testing whether Impdh1a mutations near the ATP and GTP binding spots led to hyperactivity and elevated cGMP *in vivo*. We evaluated steady-state purine and pyrimidine metabolism and metabolic flux of glucose through glycolysis and the pentose phosphate pathway (PPP). Finally, we evaluated Impdh1a filament size and localization to look for protein aggregation. Our results indicate that a hyperactive version of Impdh1 leading to elevated cGMP is likely not the cause of photoreceptor death in our models. Other changes in metabolism, Impdh1a protein polymerization and localization provided new clues regarding the cause of cell death. The availability of the animal models we generated in this study will allow further evaluation of these new hypotheses.

## RESULTS

### Zebrafish models with Impdh1a class I mutations show hyperactivity *in vitro*

**Fig. 1.**
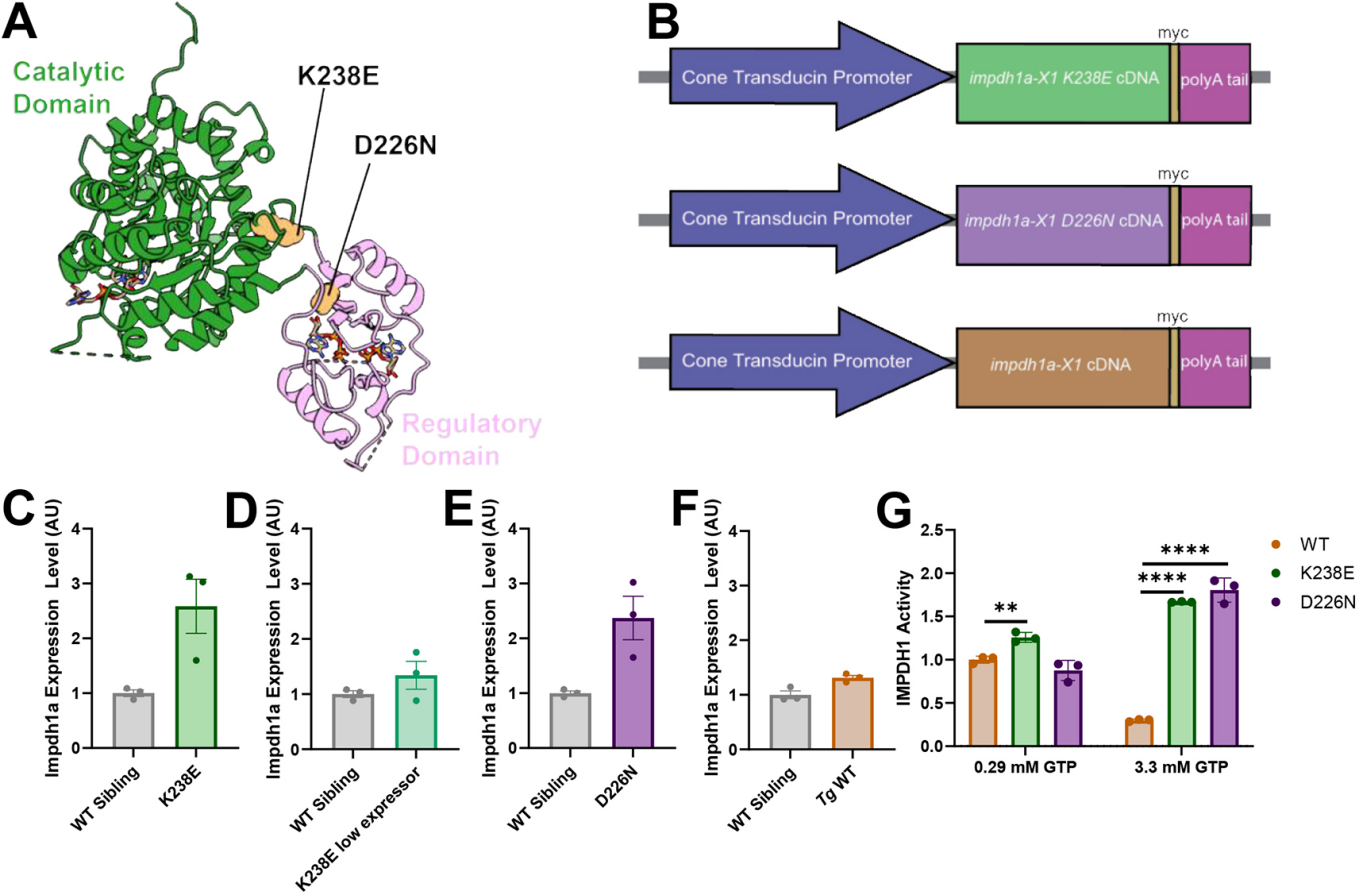
**Zebrafish *impdh1a* transgenic line generation.** (A) Two disease-associated IMPDH1 mutations (orange) on IMPDH1 monomer that were stably expressed in zebrafish. (B) Schematic describing the transgenic zebrafish lines. All lines are under a cone transducin promoter, and have a myc tag and a polyA tail. Mutant (K238E or D226N) or wild-type (WT) *impdh1a* cDNA were injected. (C) *impdh1a* expression in 7 days post fertilization (dpf) zebrafish larval eyes expressing K238E *impdh1a* mutation or WT sibling larval eyes. *n*=3 for K238E and WT siblings. Error bars are s.e.m. (D) *impdh1a* expression in 7 dpf zebrafish larval eyes expressing K238E *impdh1a* mutation or WT sibling larval eyes. *n*=3 for K238E and WT siblings. Error bars are s.e.m. (E) *impdh1a* expression in 7 dpf zebrafish larval eyes expressing D226N *impdh1a* mutation or WT sibling larval eyes. *n*=3 for D226N and WT siblings. Error bars are s.e.m. (F) *impdh1a* expression in 7 dpf zebrafish larval eyes expressing WT *impdh1a* or WT sibling larval eyes. *n*=3 for WT *impdh1a* and WT siblings. Error bars are s.e.m. (G) Purified Impdh1 protein activity assay. Protein activity in zebrafish with K238E and D226N mutations is not inhibited by high concentrations of GTP (*P*=9.0×10^9^ for K238E and *P*=5.0×10^5^ for D226N). Protein activity in zebrafish with K238E mutation is significantly higher at 0.29 mM GTP than for WT Impdh1a protein (*P*=0.003). *n*=3 for WT, K238E and D226N. **P<0.005, ****P<0.00005 (unpaired two-tailed *t*-test). Error bars are s.d. AU, arbitrary units.

We generated two zebrafish models, each with a different class I Impdh1a mutation, K238E and D226N (Fig. 1A). Mutated versions of the retinal form of the zebrafish *impdh1a* cDNA were targeted to cone photoreceptors using the cone transducin promotor, *gnat2* (Kennedy et al., 2007; Fig. 1B, Materials and Methods). We also generated a transgenic wild-type (*Tg* WT) *impdh1a* line to test whether increased *impdh1a* expression impacted our results (Fig. 1B). All transgenes contained a *myc* tag at the C-terminus to differentiate between endogenous and transgenically expressed protein (Fig. 1B). Western blots of larval eyes from the stable transgenic strains indicated that increases in expression levels ranged from 0.88× to 3.1× (Fig. 1C-F; Fig. S1A-D) compared to those for endogenous Impdh1a. We measured Impdh1a activity at various concentrations of GTP using purified wild-type (WT) and mutated zebrafish Impdh1a protein and found that both mutations eliminated the negative allosteric regulation of the protein by GTP (Fig. 1G), similar to the results observed using human IMPDH1 (Burrell et al., 2022). If Impdh1a in cones behaved similarly to purified Impdh1a, it could lead to elevated downstream products, including cGMP and GTP, which could be fatal to the cell.

**Fig. 2.**
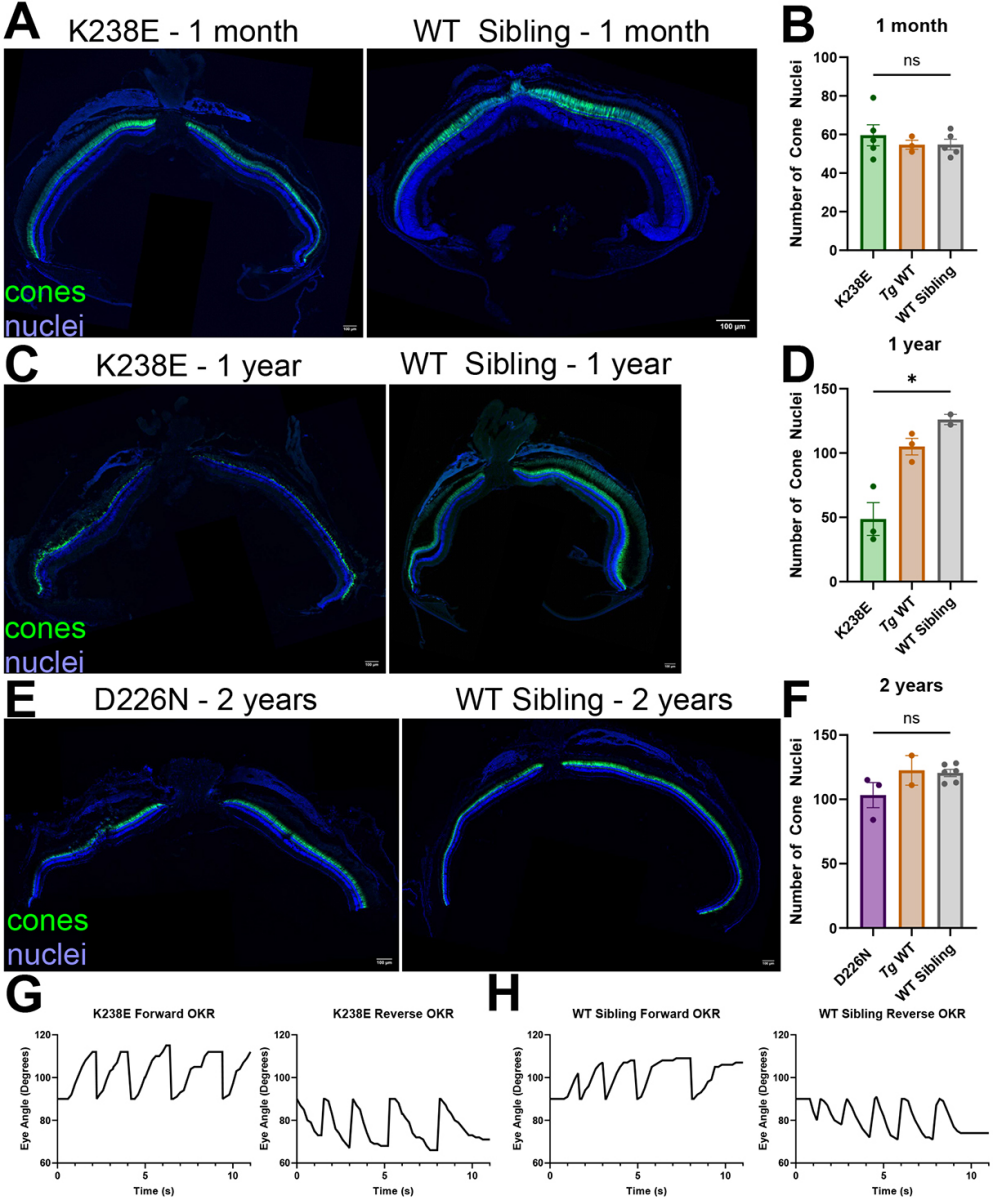
**Zebrafish with K238E mutation have severe cone degeneration by 1 year of age, whereas those with D226N mutation do not show significant signs of cone degeneration by 2 years.** (A) A 1-month-old zebrafish with K238E mutation (left) shows no signs of degeneration compared to a WT sibling (right). Cone cytosol is in green (*gnat2:EGFP* with anti-GFP antibody), and nuclei are in blue. Scale bars: 100 µm. (B) Cone nuclei were counted across one-third of the dorsal side of the retina. There was no significant change in cone nuclei for zebrafish with K238E mutation compared with those in WT siblings at 1 month. *n*=5 for K238E and WT siblings, *n*=3 for transgenic wild type (*Tg* WT). ns, not significant (*P*=0.45) (unpaired two-tailed *t*-test). Error bars are s.e.m. (C) A zebrafish with K238E mutation has significant cone loss by 1 year of age (left) compared to that in a WT sibling (right). Cone cytosol is in green (*gnat2:EGFP* with anti-GFP antibody), and nuclei are in blue. Scale bars: 100 µm. (D) Cone nuclei count of zebrafish expressing K238E IMPDH1 mutation or WT siblings. There were significantly fewer cone nuclei in zebrafish expressing K238E *impdh1a* mutation compared to those in WT siblings. *n*=3 for K238E and *Tg* WT, *n*=2 for WT sibling. *P=0.019 (unpaired two-tailed *t*-test). Error bars are s.e.m. (E) A 2-year-old zebrafish with D226N mutation (left) shows no signs of degeneration compared to a WT sibling (right). Cone cytosol is in green (*gnat2:EGFP* with anti-GFP antibody), and nuclei are in blue. Scale bars: 100 µm. (F) Cone nuclei were counted across one-third of the dorsal side of the retina. There was no significant change in cone nuclei for zebrafish with D226N mutation compared with those in WT sibling at 2 years. *n*=3 for D226N, *n*=2 for *Tg* WT and *n*=6 for WT sibling. ns, not significant (*P*=0.057) (unpaired two-tailed *t*-test). Error bars are s.e.m. (G) Optokinetic response (OKR) traces for zebrafish larvae containing K238E *impdh1a* mutation. Zebrafish larvae have forward (left) and reverse (right) OKR response at 5 dpf. (H) OKR traces for WT siblings to zebrafish larvae containing K238E *impdh1a* mutation. Zebrafish larvae have forward (left) and reverse (right) OKR response at 5 dpf.

In humans, the IMPDH1 mutations K238E and D226N cause photoreceptor degeneration (for review, see Sakti et al., 2023). To evaluate whether cones degenerate in our transgenic models, we crossed our strains with a previously established line expressing eGFP in the cone cytosol [*Tg*(*gnat2:eGFP*); (Kennedy et al., 2007)]. At 1 month of age, zebrafish with the K238E Impdh1a mutation showed no signs of cone loss (Fig. 2A,B). K238E mutant zebrafish showed signs of degeneration starting at 2 months of age and had significant degeneration at 4 months (Fig. S2A-D). Some residual cones were present up to 1 year of age (Fig. 2C,D). To verify that cone degeneration was not due to expression levels of the transgene, we evaluated cone loss in another zebrafish line with the K238E mutation expressed at lower levels (expressed at 1.3× versus 2.6×) (Fig. 1D; Fig. S2E,F). This strain also showed significant cone degeneration by 4.5 months (Fig. S2E,F). In contrast, zebrafish expressing *impdh1a* containing the D226N mutation did not have significant cone loss by 2 years of age (Fig. 2E,F; Fig. S2G,H). Transgenic WT *impdh1a* zebrafish appeared to have a subtle cone degeneration phenotype at 4 months, but we did not detect this at later timepoints (Fig. 2B,D,F; Fig. S2A-D,G-I). Prior to any degeneration, all transgenic *impdh1a* zebrafish models were sighted and had normal forward and reverse optokinetic responses (OKRs) (Fig. 2G,H; Fig. S2J,K).

### Glucose usage remains unchanged with D226N mutation, but cellular redox status is perturbed

**Fig. 3.**
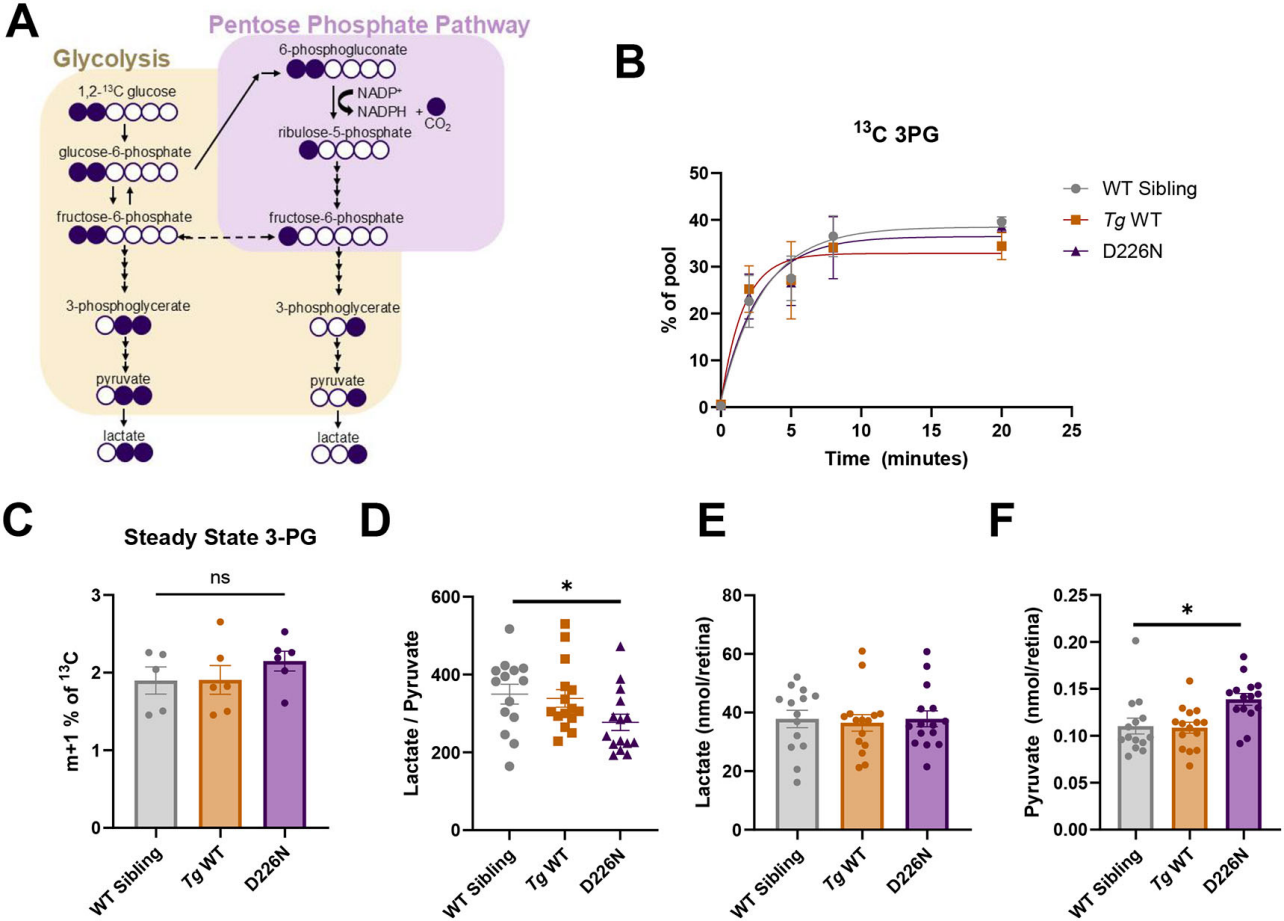
**Glucose usage remains unchanged in zebrafish with D226N Impdh1a mutation.** (A) Schematic illustrating the labeling pattern using 1,2 ^13^C glucose. If glucose goes through glycolysis, two heavy carbons remain, whereas if glucose is diverted to the pentose phosphate pathway, only one heavy carbon remains. (B) Accumulation of ^13^C 3PG over time. There are no differences in levels of labeled 3PG over time between D226N, *Tg* WT or WT sibling zebrafish retinas. *n*=3 for all time points and groups except WT sibling at 8 h time point (*n*=2). Error bars are s.e.m. (C) Steady-state levels of pentose phosphate-derived 3PG (m+1). D226N mutation did not increase glucose diversion to pentose phosphate pathway. *n*=5 for WT sibling, and *n*=6 for D226N and *Tg* WT. ns, not significant (*P*=0.27) (unpaired two-tailed *t*-test). Error bars are s.e.m. (D) Total lactate to pyruvate levels of D226N, *Tg* WT or WT sibling zebrafish retinas. Zebrafish with D226N mutation have a lower lactate to pyruvate ratio. *n*=14 for WT sibling, and *n*=15 for D226N and *Tg* WT. *P=0.036 (unpaired two-tailed *t*-test). Error bars are s.e.m. (E) Total lactate levels (nmol/retina) for D226N, *Tg* WT or WT sibling zebrafish retinas. There were no significant differences in total lactate levels. *n*=14 for WT sibling, and *n*=15 for D226N and *Tg* WT. Error bars are s.e.m. (F) Total pyruvate levels (nmol/retina) for D226N, *Tg* WT or WT sibling zebrafish retinas. Total pyruvate was higher in zebrafish with D226N *impdh1a* mutation than in WT siblings. *P=0.011 (unpaired two-tailed *t*-test). *n*=14 for WT sibling, and *n*=15 for D226N and *Tg* WT. Error bars are s.e.m.

We hypothesized that if Impdh1a were hyperactive, we would detect additional guanine nucleotides. This could require increased shunting of glucose-6-phosphate into the PPP, leading to the synthesis of more ribose-5-phosphate. To evaluate this, we measured flux through the PPP in isolated retinas incubated in 1,2 ^13^C glucose. We tested this in zebrafish retinas expressing the D226N mutation because we could reliably isolate the retina to expose it to heavy-labeled glucose. We were not able to test this in zebrafish containing the K238E mutation owing to the rapid degeneration and the requirement to use ∼30 dissected zebrafish retinas per time point at 1 month of age. When 1,2 ^13^C glucose goes through glycolysis, both heavy labels remain in the end products of glycolysis, whereas if 1,2 ^13^C glucose is shunted to the PPP, only one heavy-labeled carbon remains (Fig. 3A). There was no difference in the rate of production of ^13^C 3PG for the D226N mutant or *Tg* WT zebrafish compared to that in non-transgenic siblings (Fig. 3B). We also did not detect a difference in the steady-state amount of m+1 3PG (PPP derived) between D226N mutants or *Tg* WT compared to WT siblings (Fig. 3C). These results indicate that the amount of glucose being shunted to the PPP remains unchanged.

To convert IMP to XMP, IMPDH1 reduces NAD^+^ to NADH. Another prediction, if Impdh1 were hyperactive, is that the NADH to NAD^+^ ratio would increase. Measuring the lactate to pyruvate ratio is a way to assess the cellular NADH to NAD^+^ ratio (Williamson et al., 1967). The lactate:pyruvate ratio was significantly decreased in the D226N mutant zebrafish compared to that in WT retinas (Fig. 3D). Total lactate levels remained constant between all samples (Fig. 3E), but total pyruvate levels were significantly higher in the D226N mutant zebrafish compared to those in WT retinas (Fig. 3F). This suggests a decrease in NADH compared to NAD^+^ and provides evidence against the enzyme hyperactivity hypothesis; if Impdh1a were hyperactive, we would expect to see an increase in NADH.

### Manipulating Impdh1a activity does not significantly affect cGMP levels in the retina

**Fig. 4.**
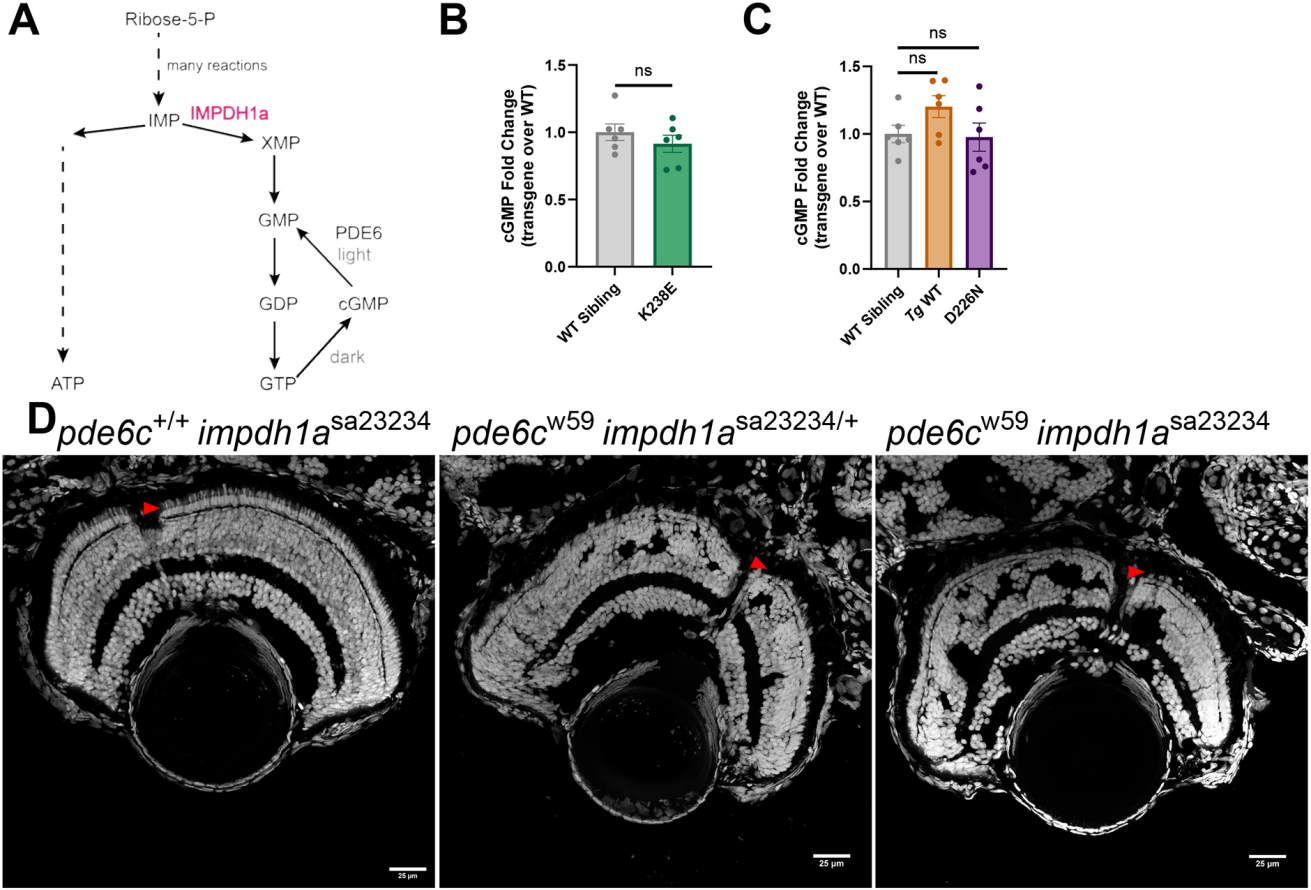
**Altering Impdh1a does not affect cGMP levels.** (A) Schematic of *de novo* purine biosynthesis of cGMP, with IMPDH1a highlighted in red. In the light, phosphodiesterase 6 (PDE6) converts cGMP into GMP. (B) Steady-state levels of cGMP from K238E mutant zebrafish or WT siblings. Zebrafish with class 1 mutant, K238E, do not have raised steady-state levels of cGMP. *n*=6 for both groups. ns, not significant (*P*=0.36) (unpaired two-tailed *t*-test). Error bars are s.e.m. (C) Steady-state levels of cGMP from *Tg* WT, D226N mutant or WT zebrafish. Zebrafish with class 1 mutant, D226N, do not have raised steady-state levels of cGMP. *n*=6 for all groups. ns, not significant (*P*=0.079 for *Tg* WT and *P*=0.85 for D226N) (unpaired two-tailed *t*-test). Error bars are s.e.m. (D) Larval optic nerve slices with nuclei stained with Hoechst. Scale bars: 25 µm. An Impdh1a KO zebrafish shows no signs of degeneration, whereas the Pde6c mutant has signs of severe degeneration at 5 dpf. The Pde6c mutant, Impdh1a KO zebrafish also shows signs of severe degeneration by 5 dpf. Knocking out Impdh1 did not rescue or delay degeneration of the model with high cGMP. Red arrowheads point to the cone layer.

cGMP is a key signaling molecule in phototransduction. It is well established that increases in cGMP lead to photoreceptor degeneration (Iribarne and Masai, 2018; Power et al., 2020). cGMP is made from GTP (Fig. 4A). We hypothesized that by manipulating the rate-limiting enzyme in *de novo* purine biosynthesis, Impdh1a, we could manipulate GTP levels and consequently alter cGMP pool sizes. Hyperactive Impdh1a could cause high steady-state levels of cGMP. To evaluate this, we used mass spectrometry to measure steady-state cGMP in retinas/eyes from *Tg* K238E, *Tg* D226N or *Tg* WT zebrafish and compared these results to those from age-matched non-transgenic retinas. We found no increase in steady-state levels of cGMP for either *Tg impdh1a* mutant or *Tg* WT retinas compared to non-transgenic zebrafish retinas (Fig. 4B,C).

In photoreceptors, cGMP concentrations are maintained by the activity of phosphodiesterase 6 (PDE6), which converts cGMP to GMP in the light (Arshavsky et al., 2002). Loss of PDE6 in either rods or cones leads to photoreceptor degeneration (Farber and Lolley, 1974; Kennedy et al., 2007; Power et al., 2020). To further evaluate whether cGMP levels could be influenced by changes in Impdh1a activity, we crossed a previously characterized Impdh1a knockout (KO) zebrafish (Cleghorn et al., 2022) with a zebrafish strain lacking the cone catalytic subunit of Pde6c (*pde6c^w59^*) (Stearns et al., 2007). Loss of Pde6c causes rapid degeneration of zebrafish cones (Stearns et al., 2007). Knocking out Impdh1a does not cause photoreceptor degeneration but does cause a 50% decrease in retinal guanine levels (Cleghorn et al., 2022). We hypothesized that the loss of Impdh1a could slow cGMP production and thus rescue the Pde6c mutant by reducing levels of cGMP. By 5 days post fertilization (dpf), cone nuclei were absent from Pde6c mutant, but not from Impdh1a KO, zebrafish as expected (Fig. 4D). However, cone loss in the Pde6c mutant zebrafish was not rescued by knocking out Impdh1a (Fig. 4D).

### Impdh1a mutations cause alteration in steady-state purine or pyrimidine metabolism

**Fig. 5.**
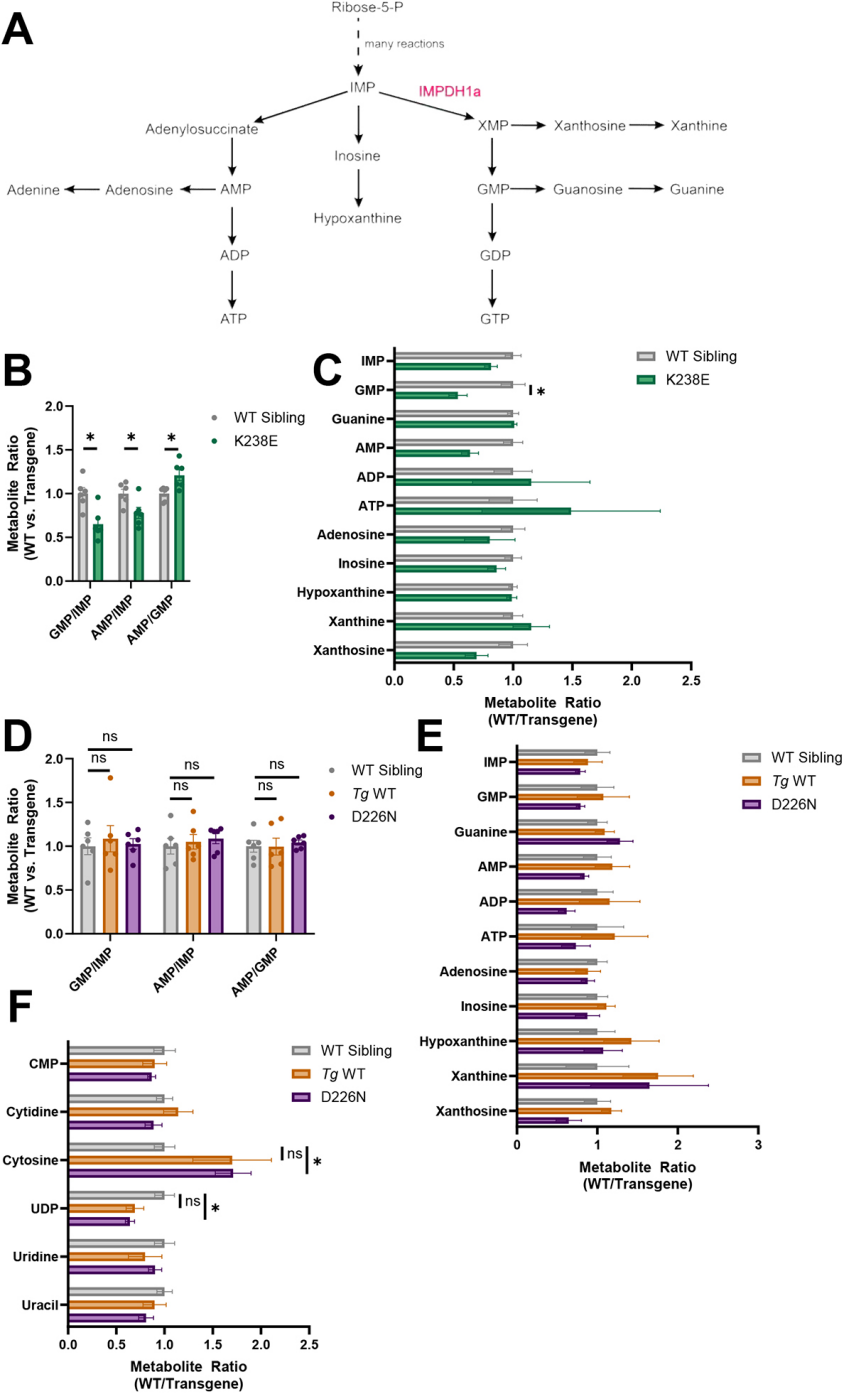
**Purine or pyrimidine steady-state metabolite levels are affected in zebrafish with Impdh1a mutations.** (A) Schematic showing *de novo* purine biosynthesis. IMPDH1a is highlighted in red. (B) Steady-state metabolite ratios in K238E mutant zebrafish compared to WT sibling eyes. GMP/IMP and AMP/IMP were lower in K238E mutant zebrafish compared to WT siblings (*P*=0.015 and *P*=0.027, respectively), whereas AMP/GMP was higher in K238E mutants (*P*=0.027). *n*=6 for both groups. Significance determined by unpaired two-tailed *t*-tests with Holm-Šídák correction for multiple comparisons. Error bars are s.e.m. (C) Ratio of purine-related metabolites for K238E mutant or WT sibling zebrafish eyes. GMP was significantly lower in K238E mutant zebrafish compared to WT siblings. *n*=6 for both groups. *P=0.048 (unpaired two-tailed *t*-tests with Holm-Šídák correction for multiple comparisons). Error bars are s.e.m. (D) Steady-state metabolite ratios in D226N mutant or *Tg* WT compared to WT sibling zebrafish retinas. Ratios in D226N mutant and *Tg* WT zebrafish retinas were not significantly different from those in WT sibling retinas. *n*=6 for all groups. ns, not significant (unpaired two-tailed *t*-tests with Holm-Šídák correction for multiple comparisons). Error bars are s.e.m. (E) Ratios of purine-related metabolites in D226N mutant, *Tg* WT or WT sibling zebrafish retinas. There were no significant differences in purine metabolites between mutant and WT retinas (unpaired two-tailed *t*-tests with Holm-Šídák correction for multiple comparisons). *n*=6 for all groups. Error bars are s.e.m. (F) Ratios of pyrimidine-related metabolites in D226N mutant, *Tg* WT or WT sibling zebrafish retinas. In retinas from D226N mutant zebrafish, cytosine was significantly higher (*P*=0.046), and UDP was significantly lower (*P*=0.049), than in WT sibling retinas, but there were no differences in cytosine (*P*=0.49) and UDP (*P*=0.26) between *Tg* WT and WT sibling retinas. *n*=6 for all groups. Significance determined by unpaired two-tailed *t*-tests with Holm-Šídák correction for multiple comparisons. Error bars are s.e.m.

To more broadly analyze whether nucleotides were altered in our transgenic lines, we next measured steady-state levels of both purine and pyrimidine metabolites. IMPDH1 converts IMP into XMP, which can be converted into GMP and other downstream metabolites (Fig. 5A). If IMPDH1 is inhibited, IMP can be shunted to make adenosine monophosphate (AMP) and its other associated products or inosine (Fig. 5A). We isolated eyes from zebrafish with the K238E mutation prior to degeneration and analyzed steady-state metabolites from the purine and pyrimidine pathways. Zebrafish with the K238E Impdh1a mutation had lower GMP/IMP and AMP/IMP ratios (Fig. 5B). The AMP/GMP ratio was higher in the K238E mutant zebrafish compared to that in non-transgenic siblings (Fig. 5B). GMP metabolite levels were significantly lower in K238E mutants compared to those in non-transgenic zebrafish (Fig. 5C). This suggests that, instead of being hyperactive, the K238E mutation causes the protein to be inactive and to dominantly inhibit the synthesis of adenine nucleotides.

To analyze purine and pyrimidine metabolites for the D226N Impdh1a mutant zebrafish, we isolated retinas from dark-adapted 3-month-old zebrafish. D226N Impdh1a-containing retinas did not show any changes in GMP/IMP, AMP/IMP or AMP/GMP ratios (Fig. 5D). *Tg* WT retinas also had no changes in these ratios (Fig. 5D). Neither the D226N Impdh1 mutant nor the *Tg* WT zebrafish had any changes in steady-state purine metabolites (Fig. 5E). Cytosine, a pyrimidine metabolite, was significantly elevated in D226N mutant retinas but trended in the same direction for *Tg* WT retinas (Fig. 5F). UDP, another pyrimidine metabolite, was decreased significantly in D226N mutant retinas and trended in the same direction for *Tg* WT retinas (Fig. 5F). Overall, the metabolic phenotype of D226N mutants appeared like that of WT.

### Cones of Impdh1a mutant zebrafish have abnormally large filaments

**Fig. 6.**
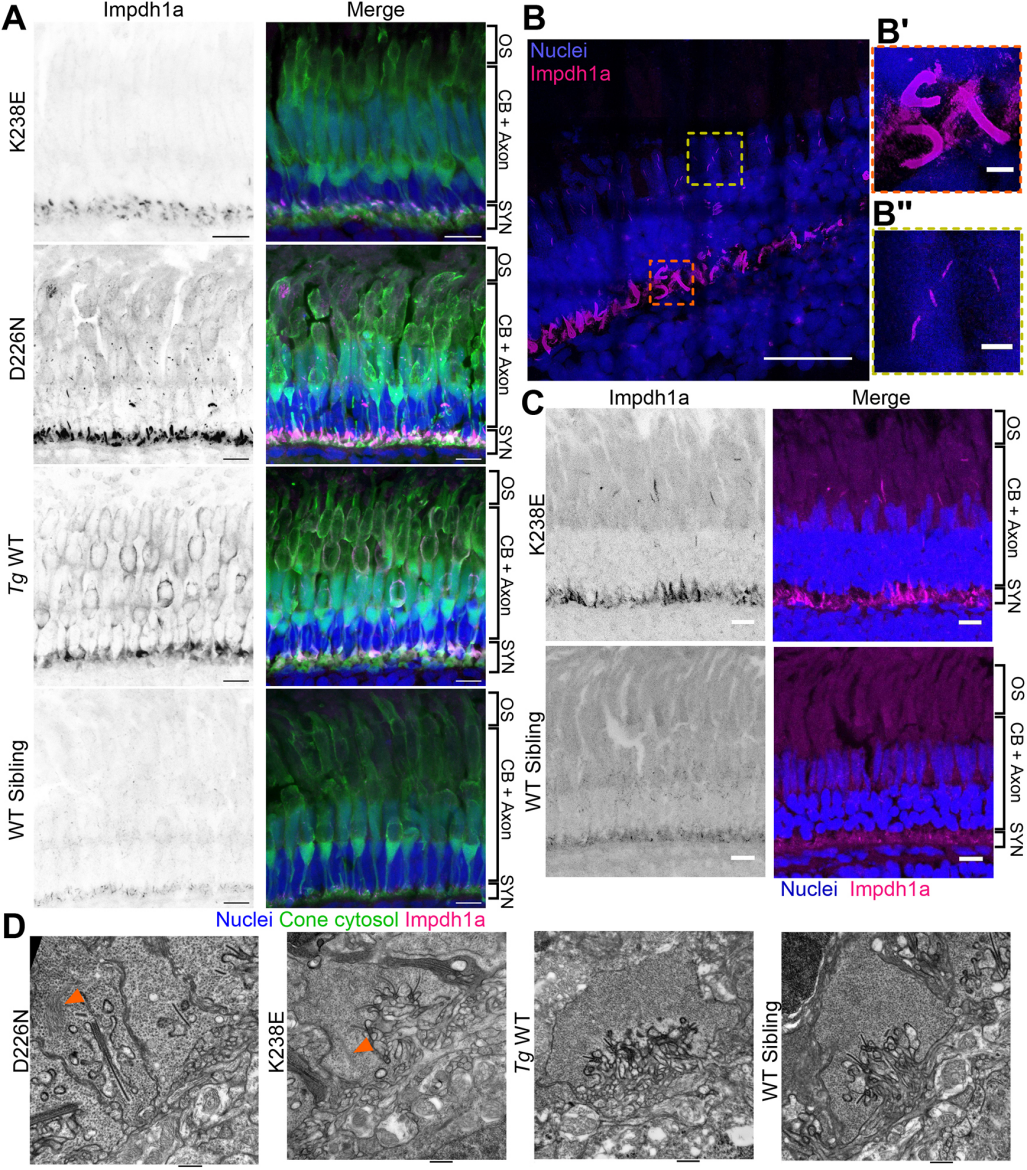
**Filaments from zebrafish with Impdh1a mutations are larger and localized to synapses (K238E) or synapses and nuclei (D226N).** (A) K238E Impdh1a mutant, D226N Impdh1a mutant, *Tg* WT or WT sibling zebrafish were crossed with *TαCP:eGFP* (cone cytosol, green), and retinal sections were stained with Hoechst (nuclei, blue) and a custom anti-Impdh1a antibody (magenta). K238E mutant zebrafish have larger filaments at cone synapses, and D226N mutant zebrafish have larger filaments at cone synapses and nuclei, whereas *Tg* WT zebrafish have diffuse cytosolic Impdh1a staining. Images show the cone layer. CB, cell bodies; OS, outer segment; SYN, synapses. Scale bars: 10 µm. (B) D226N Impdh1a mutant retinal sections expanded 5.5×. Nuclei (blue) and Impdh1a (magenta). B′ is a zoomed-in area of a cone synapse (orange dashed line box); B″ is a zoomed-in area of a Impdh1a-positive filament in cone nuclei (yellow dashed line box). Scale bars: 10 µm (B); 1 µm (B′,B″). All scale bars corrected by expansion factor (5.5). (C) K238E Impdh1a mutant (top) and WT sibling (bottom) zebrafish retinas stained with Hoechst (nuclei, blue) and a custom anti-Impdh1a antibody (magenta) at 4 months of age. The K238E mutant zebrafish retina has large filaments at the cone synapses and in cell bodies near the mitochondrial cluster, whereas the WT sibling retina has small Impdh1a filaments throughout the cell bodies and synapses. Images show the cone layer. Scale bars: 10 µm. (D) Transmission electron micrographs of cone synapses of D226N mutant, K238E mutant, *Tg* WT or WT sibling zebrafish. Zebrafish with Impdh1a D226N and K238E mutations have filamentous structures in synapses (orange arrowheads). Zebrafish eyes were taken prior to degeneration. Scale bars: 600 nm (D226N); 500 nm (others).

WT cones have small Impdh1a filaments dispersed throughout the cell body, synapse and occasionally the outer segment (Cleghorn et al., 2022). We analyzed the K238E Impdh1a mutant crossed with *Tg*(*gnat2:EGFP*) (cone cytosol marker) with a custom anti-Impdh1a antibody prior to degeneration (Fig. 6A). The K238E mutant had large Impdh1a-positive structures in cone synapses (Fig. 6A). D226N mutant zebrafish had similar large structures in the synapses and small filamentous structures by the cone nuclei (Fig. 6A). These large structures were distinct from small filaments in non-transgenic zebrafish and diffuse cytosolic signal in *Tg* WT zebrafish (Fig. 6A).

To obtain better visualization of the Impdh1a filaments, we used expansion microscopy to enlarge our D226N samples 5.5×. With better resolution of the synapses, we observed large curved Impdh1a filaments or bundles of filaments (Fig. 6B,B′). We were also able to visualize the nuclear Impdh1a filaments with greater clarity and found that they were inside the nuclei (Fig. 6B,B″). In contrast, nuclear-localized filaments were not identified previously in non-transgenic WT cones (Cleghorn et al., 2022). Impdh1a filaments were difficult to observe in 1-month expanded samples owing to size. In 4-month-old eyes of zebrafish expressing the K238E mutation, large filaments were observed at the synapse and near cone mitochondria in greater clarity (Fig. 6C) in remaining cones. Filaments were also not observed in *Tg* WT sections post expansion, supporting the idea that large Impdh1a filaments are exclusive to K238E and D226N mutants.

To determine whether Impdh1a-positive filaments could be impacting synaptic structure, we used transmission electron microscopy (TEM) to evaluate synapse integrity. Cone synapses contained ribbons, vesicles and no obvious defects (Fig. 6D); however, we noticed that in some of the mutant transgenic synapses [*n*=11 structures (104 synapses) for D226N and *n*=7 structures (58 synapses) for K238E], large filamentous structures were within the synapses (Fig. 6D). We speculate that these structures are Impdh1a positive as they were not detected in WT or *Tg* WT cone synapses.

## DISCUSSION

Photoreceptors require specific levels of cGMP for phototransduction, and cGMP imbalances can lead to photoreceptor degeneration (Farber and Lolley, 1974; Power et al., 2020). In phototransduction, light activates a G protein-coupled receptor signaling pathway in which PDE6 is activated in photoreceptor outer segments. PDE6 then hydrolyzes cGMP to GMP, which leads to the closure of CNG channels (Arshavsky et al., 2002). During recovery from light stimulation, GMP from the outer segment is converted to GDP and subsequently GTP in the inner segment of photoreceptors (Wimberg et al., 2018). Guanylyl cyclase converts GTP to cGMP in photoreceptor outer segments, thus re-opening CNG channels until phototransduction is re-activated. GTP derived from *de novo* purine biosynthesis is important for cGMP regeneration during phototransduction recovery (Plana-Bonamaisó et al., 2020). Light-induced IMPDH1 phosphorylation can increase *de novo* purine biosynthesis (Plana-Bonamaisó et al., 2020). In zebrafish retinas, Impdh1a-mediated purine biosynthesis accounts for half of the guanine pool, the nucleobase for GTP (Cleghorn et al., 2022).

Mutations in PDE6 can lead to photoreceptor degeneration due to to high levels of cGMP (Farber and Lolley, 1974; Power et al., 2020). Inhibiting IMPDH1 pharmacologically can be sufficient to delay PDE6B-induced photoreceptor degeneration stemming from elevated levels of cGMP (Yang et al., 2020). Although guanine levels are dramatically reduced in retinas from zebrafish lacking Impdh1a, Pde6c-related photoreceptor degeneration was not delayed by knocking out *impdh1a* (Fig. 4D). Yang et al. (2020) reported that the effect of the IMPDH inhibitor was not as strong in the rd1 mouse with faster photoreceptor degeneration as in rd10 mice. Similarly, in the *pde6c^w59^* mutant, it is possible that the cGMP levels are too high to be delayed by manipulating Impdh1a alone. Additionally, cGMP can be produced by recycling purine bases through the salvage pathway (Cleghorn et al., 2022). More work is needed to delineate the contributions of *de novo* purine biosynthesis and the salvage pathway in the retina during physiological and pathological states.

Purified human and zebrafish Impdh1a protein are inhibited at high GTP levels (Burrell et al., 2022), but both K238E- and D226N-expressing zebrafish displayed Impdh1a protein activity at high GTP levels (Fig. 1G). Hyperactive IMPDH1 could lead to elevated levels of downstream products including GMP and GTP (Fig. 4A). The Km of inactivated guanylyl cyclase (GC) ranges from 1.6 to 3.2 mM, and photoreceptor GTP concentrations have been estimated to be ∼2.5 mM (Peshenko et al., 2011). If GTP levels were higher owing to aberrant IMPDH1 activity, then cGMP could elevate and initiate cell death. Similar to humans with IMPDH1 mutations (Burrell et al., 2022; Burrell and Kollman, 2022), zebrafish with K238E or D226N mutations had disrupted GTP inhibition *in vitro* (Fig. 1G). Lack of Impdh1a allosteric inhibition by GTP could increase *de novo* purine biosynthesis activity, leading to increased cGMP levels. One of the starting substrates for *de novo* purine biosynthesis is phosphoribosyl pyrophosphate (PRPP). To produce PRPP, ribose-5-phosphate from the PPP is needed. If *de novo* purine biosynthesis flux was increased, we would expect glucose diversion to the PPP to generate more substrates. We did not see an increase in m+1-labeled 3-PG, indicating that PPP flux remains unchanged (Fig. 3B,C). We also observed that the NAD^+^/NADH ratio (using lactate/pyruvate as a proxy) was decreased in D226N mutant zebrafish, opposite of what we would expect if Impdh1a were hyperactive (Fig. 3D). Additionally, the steady-state levels of cGMP were not elevated for either mutant (Fig. 4B,C). These all strongly suggest that zebrafish with K238E or D226N mutations do not show increased guanine nucleotide and cGMP synthesis and that compensatory biochemical reactions can adapt and compensate for hyperactive Impdh1a.

Although we did not find evidence of Impdh1a hyperactivity *in vivo*, there were significant alterations in steady-state metabolism and Impdh1a filament polymerization and localization. Metabolites associated with *de novo* purine biosynthesis, including IMPDH1, have been known to dynamically associate into a complex coined the purinosome (Pedley and Benkovic, 2017; Zhao et al., 2015). Disruptions in protein–protein interactions or localization of these complexes could cause downstream metabolite imbalances. K238E mutant zebrafish had significantly lower GMP/IMP and AMP/IMP ratios than WT, suggestive of less *de novo* purine biosynthesis activity (Fig. 5B). This is distinct from the zebrafish Impdh1a knockout model, in which AMP/IMP levels were unchanged (Cleghorn et al., 2022). Adenylosuccinate synthetase (ADSS) and IMPDH are both enzymes in purinosomes (Zhao et al., 2015). A disruption in their interaction could explain the reduction in GMP/IMP and AMP/IMP. Zebrafish with D226N, another class I Impdh1a mutant, had normal steady-state *de novo* purine biosynthesis but abnormal levels of cytosine and UDP, metabolites in pyrimidine synthesis (Fig. 5E,F). Notably, zebrafish with *impdh1a* expressed at a higher level also trended lower for UDP and trended higher for cytosine, indicating that this effect is due to *impdh1a* expression levels (Fig. 5F). IMPDH1 is known to interact with CTP synthase in cell culture (Chang et al., 2018). A change in interaction between these two proteins would explain the alteration in steady-state cytosine with the D226N Impdh1a mutation (Fig. 5F). More work is needed to determine whether IMPDH1 interacts with other proteins, like ADSS and CTP synthase, in the retina. The metabolic differences between K238E and D226N zebrafish mutants could indicate two different mechanisms of disease. Additionally, IMPDH1 phosphorylation can impact enzyme activity (Calise et al., 2024; Plana-Bonamaisó et al., 2020). Thus, large Impdh1a-positive structures may have altered activity due to a change in phosphorylation.

IMPDH1 also has moonlighting roles that could be causative of photoreceptor degeneration. IMPDH1 can interact with single-stranded DNA, and, when expressing D226N in cell culture, the affinity of IMPDH1 for single-stranded DNA decreases (Mortimer and Hedstrom, 2005). IMPDH1 can also interact with polyribosomes translating rhodopsin mRNA (Mortimer et al., 2008). However, it is unclear whether IMPDH1 mutations disrupt this interaction and whether this disruption would lead to a change in rhodopsin protein levels. Further, mice with a single rhodopsin gene only have a mild retinopathy (Lem et al., 1999).

It is unclear why D226N mutant zebrafish cones did not degenerate. One explanation may be that this mutation in humans preferentially affects rods and not cones; another possibility is that zebrafish cones are somehow protective against the effects of this mutation. Either possibility would highlight important information regarding the mechanism of photoreceptor cell death. Because we did not examine the visual responses of cones of adult zebrafish with D226N mutation, it is also possible that their function is altered. Overall, our study quantifies the biochemical consequences of IMPDH1 mutations *in vivo*, and our findings suggest that it is protein–protein interactions and/or filament size and location that could contribute to the disease caused by these mutations.

## MATERIALS AND METHODS

### Zebrafish maintenance

The University of Washington (UW) Institutional Animal Care and Use Committee authorized this research. Zebrafish were kept on a 14 h/10 h light/dark cycle at 27.5°C at the UW South Lake Union aquatics facility. Zebrafish were kept in the Roy/AB or AB background for experiments. Unless otherwise noted, zebrafish between 1 and 12 months of age were used for experiments (prior to degeneration). When possible, a mixture of male and female zebrafish were used for experiments.

### Transgenic line generation

Mutations were introduced into WT *impdh1a_tvX1* (XM_005159007) in pCR8 (Invitrogen) using the primers D306NmutF (5′-ctcggttcttcttcaaattggtacgagcgattattgc-3′), D306NmutR (5′-gcaataatcgctcgtaccaatttgaagaagaaccgag-3′), K318EmutF2 (5′-gtttgcgggagtcttcggaggccagaggata-3′) and K318EmutR2 (5′-tatcctctggcctccgaagactcccgcaaac-3′). D306N and K318E in *impdh1a tvX1* are equivalent to D226N and K238E in human IMPDH1 (NM_001142573.2). Constructs were generated using Gateway-Tol2 assembly. Assembly of the expression vector was done by combining the WT or mutated *impdh1a* pCR8 plasmid, an entry vector containing the previously characterized *gnat2* promoter (Kennedy et al., 2007) and a bleeding heart containing destination vector (Williams et al., 2010). Constructs were injected into one-cell embryos with Tol2 transposase mRNA as previously described (Kennedy et al., 2007), and three transgenic zebrafish lines were generated: *Tg*(*gnat2:impdh1a-X1-K238E*), *Tg*(*gnat2:impdh1a-X1-D226N*) and *Tg*(*gnat2:impdh1a-X1*). Single-insertion carriers from the F2 generation and beyond were used in this study. Each new generation was evaluated for continued transgene expression. The *Tg*(*gnat2*:GFP) zebrafish line was established previously (Kennedy et al., 2007).

### *In vitro* protein assays

Purified Impdh1a-X1 was diluted in 20 mM Hepes, 100 mM KCl, 1 mM dithiothreitol pH 7.0, 1 µM Impdh1a-X1, 1 mM ATP, 1 mM IMP and 300 µM NAD^+^, and various GTP concentrations were added to a 96-well UV-transparent plate. Using a Varioskan Lux microplate reader (Thermo Fisher Scientific) kept at 25°C, NADH production was measured at 340 nm by optical absorbance. One measurement/min was collected for 15 min, and absorbances were correlated with NADH concentrations generated from a standard curve. Measurements were performed in triplicate.

### Western blotting

Twenty whole eyes were enucleated from 7 dpf zebrafish larvae. Samples were homogenized on ice and then left on a shaker at 4°C for 30 min. Samples were spun down at maximum speed in a microfuge for 15 min, and then the supernatant was collected, and 12-20 μg of protein was added to each lane. Blots were probed with a custom rabbit anti-Impdh1 antibody (1:500), mouse anti-myc antibody (1:500; Cell Signaling Technology, 9B11) and anti-beta-actin antibody (1:1000; Abcam, ab8226) as previously described (Bisbach et al., 2020; Cleghorn et al., 2022). Blots were washed and incubated in secondary antibodies for 1 h prior to developing. Secondary antibodies were IRDye 680RD Donkey anti-Rabbit IgG (1:5000; Li-Cor, 926-68073) and IRDye 800CW Goat anti-Mouse IgG (1:5000; Li-Cor, 926-32210). The ladder used was Precision Plus Protein Dual Color Standards (Bio-Rad, 1610374) or Precision Plus Protein All Blue Prestained Protein Standards (Bio-Rad, 1610373). The signal intensity of the Impdh1a band was divided by the signal intensity of the actin band. WT was normalized to 1, and the transgenic line value was subtracted by 1 to account for WT expression of Impdh1a.

### Immunohistochemistry (IHC)

Eyes were enucleated and placed in 4% paraformaldehyde (PFA) fix at room temperature (RT) for 10 min. Holes were poked through the sclera, and eyes were kept in PFA for an additional 2 h at RT before being moved to a 4°C fridge for 48 h. Eyes were then sucrose protected overnight and embedded in OCT compound (Tissue-Tek, 4583). Eyes were sectioned at 14-20 µm and warmed on a slide warmer for 2 h. Sections were incubated in IHC PBS for 10 min and then blocked with normal donkey serum-based blocking buffer for 30 min. Samples were then left overnight to incubate in primary antibodies at 4°C [anti-EGFP (1:5000; Abcam, ab12970), custom anti-Impdh1 antibody (1:500; Cleghorn et al., 2022)]. Primary antibodies were removed the following day, and slides were washed three times with IHC PBS. Secondary antibodies were added (1:2000) for 1 h at RT. Secondary antibodies used included Goat anti-Chicken 488 (Invitrogen, A11039) and Goat anti-Rabbit 633 (Invitrogen, A21071). Slides were washed twice with IHC PBS and incubated with Hoechst 33342 (Invitrogen, H1399) for 10 min at RT. Sections were dried and sealed with Fluoromount G and nail polish. Slides were imaged on a Leica SP8 confocal microscope. Images were processed using ImageJ.

### Nuclei quantification

A third of the dorsal side closest to the optic nerve was used for quantification. Prior to quantification, the image was straightened, and the nuclear channel was set to grayscale. Cone nuclei were quantified using the number marker tool in ImageJ.

### OKR

Equipment comprised of a variable speed motor (Model LV3607; Electro Motor and Control Corporation), a Wild M3Z stereomicroscope with a bottom and ring light source, a camera attached on top, and a plastic drum mounted directly above the base light source made of smooth plastic lined with paper containing alternating vertical black and white stripes (∼1 cm in width), with a circular base and a motor pulley sitting 26 cm from the center of the drum (Brockerhoff, 2006). The camera was attached to a TV monitor, and an iPhone 12 was positioned on a tripod in front of the monitor to record OKRs. Methylcellulose was aliquoted into a 30 mm×15 mm Petri dish with 5-day-old larvae placed into the reagent one at a time. Fish were positioned upright and toward the edge of the Petri dish for better visualization of the lined paper. This apparatus was then placed underneath the stereoscope for observation. OKR was recorded with the iPhone for 10-20 s. The experiment was repeated to observe zebrafish OKRs in the reverse direction of the lined paper. For D226N mutant zebrafish, continuous videos of 20-30 s were taken with a direction switch at ∼10-15 s.

### OKR quantification

The most active eye was chosen for OKR measurements, which were done manually by overlaying the video on a transparent protractor image. Changes in the angle of the eye were measured for each video frame (every 0.2 s). Clockwise stripe rotations were designated to be ≥90°, and the counterclockwise direction was designated as ≤90°.

### TEM

TEM was conducted as previously described (Hutto et al., 2020).

### Expansion microscopy

Twenty-micrometer cryosections were first stained via IHC. Then, following 1 h blocking (5% normal donkey serum, 1% bovine serum albumin, 1% Triton X-100 in phosphate buffer, pH 7.4), sections were incubated overnight at RT in primary antibodies diluted in blocking buffer [rabbit anti-Impdh1aN (1:400; Cleghorn et al., 2022)]. Following three washes with phosphate buffer, sections were incubated for 2 h at RT in secondary antibodies [goat anti-rabbit AlexaFluor 546 (1:50; Invitrogen, A11071) and Hoechst 33342 (1:10; Invitrogen, H1399)]. Following three washes with phosphate buffer, sections were incubated overnight at RT in 0.1 mg/ml acryloyl X-SE in MES buffer, pH 6.0. Sections were washed twice with phosphate buffer, then incubated overnight at RT in inactivated monomer solution (7.5% sodium acrylate, 2.5% acrylamide in MOPS buffer, pH 7.0) and washed twice again with phosphate buffer. Subsequent steps of gelation, digestion and expansion were carried out as described by Asano et al. (2018), using the proExM protocol for intact tissues. Expanded gels were immobilized using super glue and a harp (Warner Instruments), and imaged in water. *Z*-stack images were acquired using a Leica SP8 confocal microscope with a 40× water dipping lens and LAS-X acquisition software. Presented images were deconvolved using Leica Lightning and maximum intensity projected.

### Liquid chromatography–mass spectrometry (LC/MS) sample collection and processing

Zebrafish were starved overnight and dark adapted for 1 h prior to dissections. Ten whole eyes (1-month samples) or four retinas (3.5-month samples) were removed and flash frozen. Retinas or eyes were homogenized in cold 80% methanol, stored on dry ice for 30 min and centrifuged at 20,817 ***g*** at 4°C for 15 min. The supernatant was dried down and reconstituted in mobile phase (30:70 in v/v of A:B) for targeted metabolomics as reported (Saravanan et al., 2023). Metabolite extracts were analyzed by a Shimadzu LC Nexera X2 UHPLC coupled with a QTRAP 5500 LC-MS/MS (Ab Sciex) with an ACQUITY UPLC BEH Amide analytic column (Waters Corp). The mobile phase was (A) 10 mM ammonium acetate (pH 8.9) in water and (B) 95/5 acetonitrile/water with 10 mM ammonium acetate (pH 8.2). The injection volume was 5 µl, and the flow rate was 0.5 ml/min, with a total run time of 11 min. The gradient elution was 95% to 61% B at 6 min, 61% to 44% B at 8 min, 61% to 27% B at 8.2 min, and 27% to 95% B at 9 min. The column was equilibrated with 95% B at the end of each run. The source and collision gas were N_2_. The ion source conditions in positive and negative mode were as follows: curtain gas, 25 psi; collision gas, high; ion spray voltage, 3800/−3800 V; temperature, 500°C; ion source gas 1, 50 psi; and ion source gas 2, 40 psi. Each metabolite was tuned with standards for optimal transitions. Nicotinamide-D4 (Cambridge Isotope Laboratories) was used as an internal standard. The extracted multiple reaction monitoring peaks were integrated using MultiQuant 3.0.3 software (AB Sciex).

### Gas chromatography–mass spectrometry (GC/MS) sample collection and processing

Zebrafish were dark adapted for 1 h prior to collection. Retinas were dissected in Krebs-Ringer buffer with 5 mM glucose added. Retinas were then moved into 1,2 ^13^C glucose and kept in an incubator at 37°C with 5% CO_2_ for various timepoints. Metabolites were extracted by adding 80% methanol and sonicated. Samples were set on dry ice for 45 min to precipitate metabolites and then spun down at 17,000 ***g*** for 30 min at 4°C. Supernatant was collected and dried down. Samples were derivatized by adding 10 µl of 20 mg/ml methoxyamine HCl in pyridine. Samples were incubated for 90 min in a 37°C oven. Then, 10 µl of TBDMS was added and put on a heating block set to 70°C for 1 h. Samples were run on an Agilent 5985 GC-MS, processed with MSD ChemStation software (Agilent) and corrected with IsoCor software (Millard et al., 2012).

## Supplementary Material

10.1242/dmm.052389_sup1 Supplementary information
