# Supplementary material for: Retinopathy-associated inosine monophosphate dehydrogenase 1 mutations cause metabolic and filament defects in cones
Source: Dis Model Mech. 2025 Sep 17;18(10):dmm052389. doi: 10.1242/dmm.052389 (PMC12486205; doi:10.1242/dmm.052389)
Supplement: Supplementary information [file dmm-18-052389-s1.pdf]

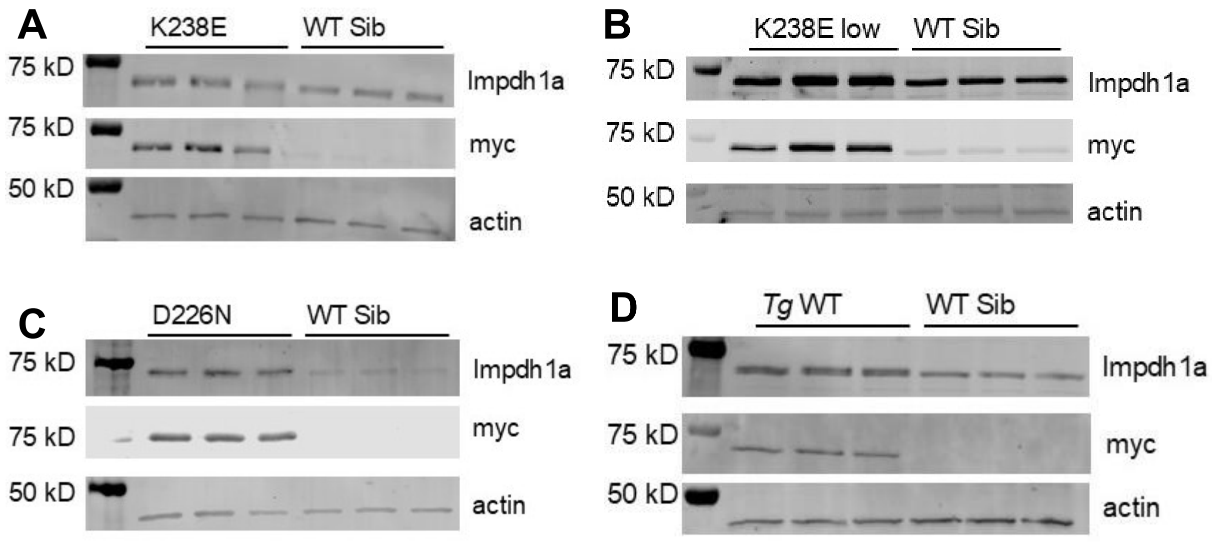

Fig. S1. Expression levels of *impdh1* transgenic lines range from 0.88 – 3.1x.

A. Western blot probed with custom Impdh1 antibody, myc antibody, and beta actin (loading control). K238E transgenic larvae are in lanes 2-4 and WT siblings are in lanes 5-7 (all 7 dpf).

B. Western blot probed with custom Impdh1 antibody, myc antibody, and beta actin (loading control). K238E transgenic larvae are in lanes 2-4 and WT siblings are in lanes 5-7 (all 7 dpf).

C. Western blot probed with custom Impdh1 antibody, myc antibody, and beta actin (loading control). D226N transgenic larvae are in lanes 2-4 and WT siblings are in lanes 5-7 (all 7 dpf).

D. Western blot probed with custom Impdh1 antibody, myc antibody, and beta actin (loading control). WT Impdh1 transgenic larvae are in lanes 2-4 and WT siblings are in lanes 5-7 (all 7 dpf).

Disease Models & Mechanisms • Supplementary information

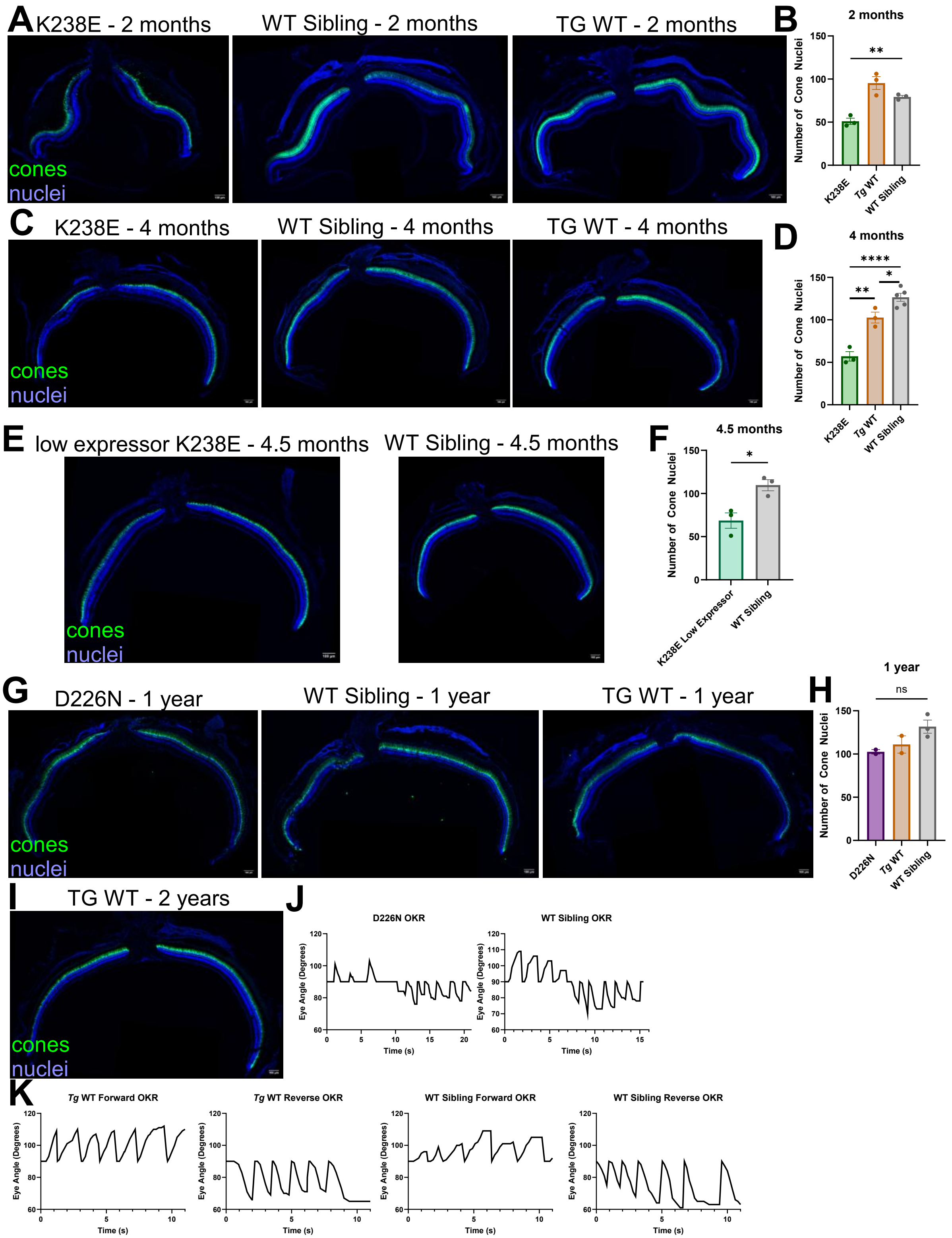

Disease Models & Mechanisms • Supplementary information

Fig. S2. K238E mutants have early signs of degeneration and D226N have no signs of cone loss at one year and normal larval visual function.

A. K238E has significant cone loss at 2 months old (left) as compared to wild type sibling (center). *Tg* WT (right) shows no sign of cone loss. Cone cytosol is in green (*gnat2:EGFP* with GFP antibody) and nuclei are in blue. Scale bar = 100μm.

B. Cone nuclei were counted across 1/3 of the dorsal side of the retina. There was significant cone loss for K238E compared wic th WT sibling at 2 months, but not with *Tg* WT. n = 3 for all groups. ** = p = 0.0021 as determined by unpaired t test. Error bars are s.e.m.

C. K238E has significant cone loss at 4 months old (left) as compared to wild type sibling (center). *Tg* WT (right) shows no sign of cone loss. Cone cytosol is in green (*gnat2:EGFP* with GFP antibody) and nuclei are in blue. Scale bar = 100μm.

D. Cone nuclei were counted across 1/3 of the dorsal side of the retina. There was significant cone loss for K238E compared with WT sibling at 4 months, but not with *Tg* WT. n = 3 for K238E and *Tg* WT and n = 5 for WT sibling. * = p = 0.022; ** = p = 0.0057; **** = p = 8.9×10^{5} as determined by unpaired t test. Error bars are s.e.m.

E. Zebrafish with K238E expressed at a lower level has significant cone loss at 4.5 months old (left) as compared to wild type sibling (right). Cone cytosol is in green (*gnat2:EGFP* with GFP antibody) and nuclei are in blue. Scale bar = 100μm.

F. Cone nuclei were counted across 1/3 of the dorsal side of the retina. There was significant cone loss for zebrafish containing lower expressed K238E compared with WT sibling at 4.5 months. n = 3 for both groups. * = p = 0.021 as determined by unpaired t test. Error bars are s.e.m.

G. 1 year old zebrafish with D226N mutation (left) show no signs of degeneration as compared to wild type sibling (center). *Tg* WT (right) shows no signs of cone loss. Cone cytosol is in green (*gnat2:EGFP* with GFP antibody) and nuclei are in blue. Scale bar = 100μm.

H. Cone nuclei were counted across 1/3 of the dorsal side of the retina. There was no significant change in cone nuclei for D226N compared with WT sibling at 1 year. n = 2 for D226N and *Tg* WT and n = 3 for WT sibling. ns = not significant (p = 0.062) as determined by unpaired t test. Error bars are s.e.m.

I. *Tg* WT zebrafish at 2 years old show no signs of cone degeneration. Cone cytosol is in green (*gnat2:EGFP* with GFP antibody) and nuclei are in blue. Scale bar = 100µm.

J. Zebrafish larvae containing D226N *impdh1* mutation OKR trace (right) and WT sibling (left). Zebrafish larvae have forward and reverse OKR response at 5dpf.

K. Zebrafish larvae containing endogenous (WT sibling) or transgenic WT IMPDH1 (*Tg* WT) OKR trace. Zebrafish larvae have forward and reverse OKR response at 5dpf.
